# Repetition costs in task switching are not equal to cue switching costs: evidence from a cue-independent context

**DOI:** 10.1007/s00426-023-01904-x

**Published:** 2023-12-19

**Authors:** Elena Benini, Iring Koch, Andrea M. Philipp

**Affiliations:** Chair of Cognitive and Experimental Psychology, Institute of Psychology, RWTH Aachen University, Jaegerstr. 17-19, 52066 Aachen, Germany

## Abstract

Recent task-switching studies highlighted the presence of feature binding processes. These studies documented that even a task-irrelevant feature (the context, henceforth) may be bound with the task and the response in each trial. When the context repeated in the following trial, it supposedly retrieved the bound features, causing benefits when the task and the response repeated and costs otherwise (i.e. full repetition benefits). In the present study, we aim to rule out an alternative explanation for such full repetition benefits in task switching. These benefits were observed in studies that used a cue-related context so that full repetition conditions always implied a cue repetition. Therefore, these full repetition benefits may be ascribed to the priming of cue encoding, instead of the binding of the context. In the present study, we implemented a similar context manipulation but used univalent target stimuli and did not present any cue. Hence, the varying context was never cue-related. We still found full repetition benefits but only when the context appeared before the target and not when they appeared simultaneously. Thus, full repetition benefits can be observed in the absence of priming of cue encoding. However, the context must occupy a prominent position (i.e. at the beginning of the trial). These results, therefore, reinforce the hypothesis that full repetition benefits stem from binding processes that take place on a trial-by-trial basis and involve both task-relevant (the task and the response) and task-irrelevant features (the context).

## Introduction

Task switching is used to measure the processes underlying humans’ goal-directed actions (e.g. Kiesel et al., 2010; Koch et al., 2018a, b). During goal-directed actions, not only goal-specific processes take place, but also, for example, automatic feature binding (Altmann, 2011; Grange et al., 2017). In the present study, we demonstrated binding processes affecting task-relevant, but also task-irrelevant features. Importantly, such task-irrelevant features were not part of any task-relevant stimuli so they could be ignored. Consequently, the present study shows that automatic binding processes involve all features of an event, even when these features are unrelated to any task-relevant stimuli and responses.

In task switching, participants switch back and forth between performing one or the other task and, thus, they maintain in working memory two task sets (e.g. Rogers & Monsell, 1995), namely, instructions about the task-relevant dimensions of the stimuli and how to respond (see Kiesel et al., 2010; Koch et al., 2018a, b, for reviews on task switching). Performing the same task as in the last trial causes very robust task repetition benefits compared to switching the task; these benefits may reflect the need, in task switches, to reconfigure the correct task (Rogers & Monsell, 1995) and/or to solve the interference carried over by the previously-performed task (Allport & Wylie, 1999; Allport et al., 1994).

Several task-switching studies showed that people can learn associations between targets and tasks (e.g. Koch et al., 2005; Pfeuffer et al., 2017; Waszak et al., 2003) and even between tasks and task-irrelevant stimuli (e.g. Rubin & Koch, 2006). Such learning can occur via contingencies, for example, when a certain target is systematically presented in combination with a certain task (Waszak et al., 2003). Participants’ performance then improves when, in a given trial, the current combination of the task and the stimulus matches with the contingent combination. In their trial sequences, Rubin and Koch (2006) created contingencies between a certain task and the colour of an irrelevant box that worked as the target’s background. Participants’ performance then decreased dramatically when these contingencies were reversed. Such results suggest that participants formed context-based expectations about a certain task given a certain contextual feature, arguably because they learned to associate things that are presented contingently.

But what are the trial-by-trial mechanisms that allow us to learn and strengthen these associations? A possibility is that such associations are formed in each trial, and then those which get repeated more often “survive” and are learned, while the others are cancelled out across trials or simply decay over time (cf. Logan, 1988 and the computational model by Schmidt, et al., 2020a, b). Such short-term associations, or bindings, are indeed assumed by the Theory of Event Coding (TEC; Hommel et al., 2001). TEC postulates that features of a certain segment of experience (i.e. an event) are automatically bound together and such bindings are retrieved whenever an element of a binding re-occurs. Such features include both perceptual and action features that are represented in the brain via a common coding (Prinz, 1990). When a feature repeats from the previous trial, and thus retrieval takes place, performance improves if all the features repeat as well (i.e. a full repetition) compared to when some feature switches (i.e. a partial repetition). Such improvement (i.e. full repetition benefits) might be due to the retrieved features priming the current features (Henson et al., 2014), and/or to the absence of any interference between the retrieved event and the current trial (Altmann, 2011; Frings et al., 2020; Mocke et al., 2023). Finally, when no feature repeats (i.e. a full switch), the previous trial episode is not retrieved thus this condition should yield (1) full switch benefits compared to partial repetitions on the one hand, as there is no retrieval interference, but (2) no repetition priming benefits, thus worse performance compared to full repetitions on the other hand. Taken together, binding and retrieval processes leave behind full repetition benefits (and, sometimes, full switch benefits too), as an empirical marker. Investigating trial-by-trial associative processes may thus help in understanding the short-term mechanisms that may constitute the building blocks of longer-term learning processes.

**Fig. 1:**
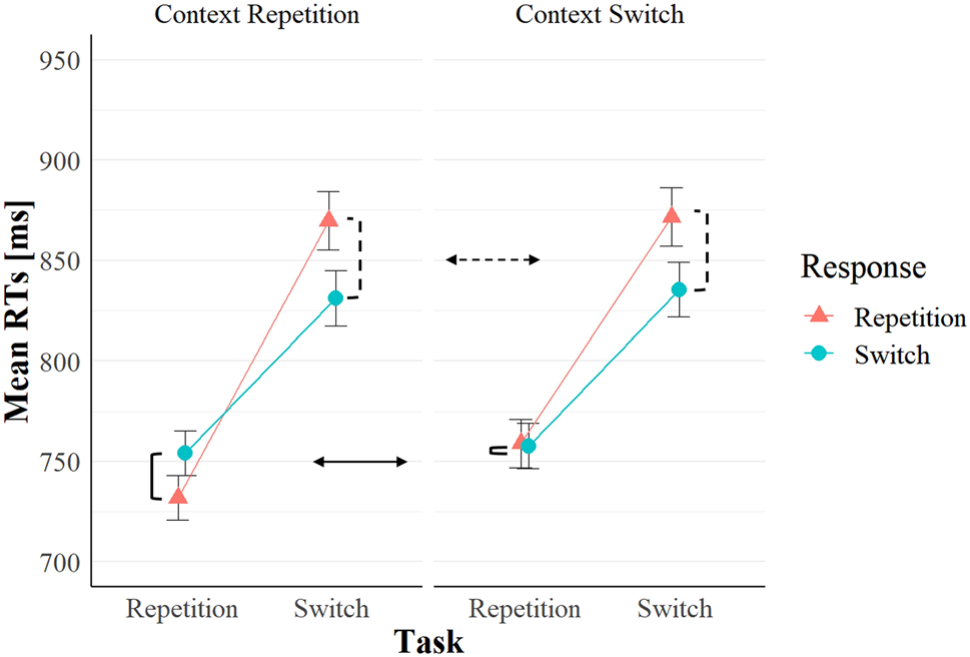
Results of Experiment 1 of Benini et al. (2023). The full repetition benefits are visible when comparing the RR benefits (solid square brackets) in context repetitions (left panel) versus switches (right panel). The absence of full switch benefits emerges instead by comparing the RR costs (dotted square brackets) in the left versus the right panel. Data of 124 participants. The context was the task-irrelevant colour of the cue rectangle, and the orientation of the rectangle indicated the task to perform. Reaction times are averaged across two SOAs levels. Vertical bars indicate standard errors of the means

In task switching, repeating the response often causes benefits in task repetitions, but costs in task switches. This interaction between task *n*–1 transition and response *n*−1 transition is often called the response-repetition (RR) effect (e.g. Altmann, 2011; Druey, 2014; Hübner & Druey, 2006; Kleinsorge, 1999). Interestingly, the RR effect may reflect short-term task-response binding (Altmann, 2011; Koch et al., 2023; Koch et al., 2018a, b, see also Schmidt, et al., 2020a, b for a computational model that can simulate the RR effect by modelling a memory storage where elements of each trial are stored in a memory trace that can be retrieved and see Druey, 2014; Gade et al., 2014, for an overview of competing theoretical accounts). Indeed the RR effect entails both full repetition benefits and full switch benefits (see the left panel of Fig. 1).

## The context-binding hypothesis

Recent studies (Benini et al., 2022, 2023; Kandalowski et al., 2020; Koch et al., 2018a, b) built on the binding hypothesis for the RR effect and investigated whether an additional, task-irrelevant feature is bound with the task and the response in task switching. Such task-irrelevant feature (the context, henceforth) was never contingent to any task, response or target, differently from task-switching studies examining how contingencies are learnt. In fact, in a given trial, both features from the n−1 trial and features associated via longer-term contingency learning might be retrieved. The effect of such diverse retrievals would be difficult to disentangle and would increase noise in the data. Therefore, contingencies are usually avoided when the focus is on short-term bindings.

In most of the studies mentioned above, the context was often cue-related (i.e. cue modality, colour or language, depending on the experiment), it was irrelevant to the task and could switch or repeat in each trial like the task and the response. Often, there were smaller (and sometimes null) RR benefits in task repetitions when the context switched compared to when it repeated (see Fig. 1, data referring to Benini et al., 2022a). Statistically, context transition (repetition vs. switch) interacted with task transition and response transition. Despite the interaction was not always significant (i.e. in Kandalowski et al., 2020 and in Benini et al., 2022b, Experiment 2), the pattern looked consistent across the studies. In other words, the full repetition condition yielded better performance than a partial repetition condition in which the task and the response repeated, but the context switched. Such full repetition benefits then support the notion that the context is bound with the task and the response and retrieves them when it repeats, but not when it switches (we refer to this as the *context-binding hypothesis* henceforth).

The RR costs in task switches were instead unaffected by repeating versus switching the context (see Fig. 1). However, feature-binding accounts would predict full switch benefits. In the cited studies, performance in the full switch condition, namely when the context, the task and the response switched, was not better than in the partial repetition condition (i.e. when the context switched, but the task and the response repeated), since switching the context did not increase RR costs in task switch trials (and in Experiment 2 of Benini et al., 2022a, switching the context even *decreased* them). The absence of full switch benefits represents a challenge to the context-binding hypothesis, however, is consistent with an alternative hypothesis, namely *priming of cue encoding* (see the next paragraph).

## The priming of cue encoding hypothesis

This alternative hypothesis suggests that *priming of cue encoding* (Schneider & Logan, 2005; see also Monsell & Mizon, 2006) might be responsible for full repetition benefits in the above-mentioned task-switching studies. Priming of cue encoding is a specific case of a repetition priming process, whereby reaction to a pre-exposed stimulus (i.e. reaction to the cue, in these studies) is faster than to a novel one (Neill, 1997).

The concept of priming of cue encoding was put forward to explain why, in task switching, repeating the cue causes larger task repetition benefits compared to switching it (Logan & Bundesen, 2003; Mayr & Kliegl, 2003; see Jost et al., 2013, for a review). This pattern emerges in studies in which each task is cued by two different cues, often called 2:1 cue:task mapping studies. The cue is a stimulus typically presented before the target that indicates the task to perform in each trial. With two cues per task, there are (1) cue repetitions when both the task and the cue repeat, (2) task repetitions when the task repeats, but the cue switches, and (3) task switches when the task switches, and thus the cue switches, too. Typically, not only task repetitions are faster than task switches, but also cue repetitions are faster than task repetitions (e.g. Monsell & Mizon, 2006). According to the priming of cue encoding hypothesis, this pattern is due to the faster processing of a cue when this cue was already processed in the previous trial (i.e. priming; Schneider & Logan, 2005).

Superficially, such a design is similar to the above-mentioned task-switching studies that introduced a cue-related context (e.g. cue colour, or cue modality). Indeed, having a cue-related context implies having two different versions of the same cue (e.g. blue vs. red, or visual vs. auditory), thus, arguably, two cues per task. Thus, in task repetitions, there are cue repetitions when also the context repeats and task repetitions when the context instead switches. Consistent to 2:1 cue:task mapping studies, cue-related context studies found better performance when both the task and the context repeated compared to when the task repeated but the context switched (if averaging across the response repetition versus switch factor, see Fig. 1). The main difference between 2:1 cue:task mapping studies and cue-related context studies emerges instead in task switches: while a cue-related context can repeat when the task switches, no cue repetition is possible in 2:1 cue:task mapping studies. In other words, in cue-related context studies, context repetition/switch is orthogonal to task repetition/switch; however, in 2:1 cue:task mapping studies, the cue can repeat or switch when the task repeats, but it must switch when the task switches.

Hence, how could priming of cue-encoding explain the results of context-related studies, if the designs differ? The reason is that the effect of context transition was visible only in task repetitions, where the two designs overlap and yield consistent results (i.e. cue/context repetitions are faster than task repetitions), while there is typically no effect of context transition in task switches, where the two designs differ. Therefore, ignoring the response repetition factor, the effect of repeating a cue-related context is very similar to the effect of repeating a cue in 2:1 cue:task mapping studies.

Concerning the response repetition factor, there is no consensus on whether cue repetitions augment *RR benefits* in task repetitions. To our knowledge, very few task-switching studies explicitly assessed RR benefits in task repetitions as a function of *cue* transition and found smaller (Altmann, 2011; Schmidt & Liefooghe, 2016), or no RR benefits with cue switches (Arrington & Logan, 2004; Schneider & Logan, 2005; but see Mayr & Kliegl, 2003). Thus, repeating the cue may increase RR benefits in task repetitions. Therefore, results obtained with a cue-related context are compatible with the results of 2:1 cue:task mapping studies also when the response repetition factor is taken into account.

Interestingly, the fact that repeating the cue increases RR benefits in task repetitions might also reflect binding effects (cf. Schmidt & Liefooghe, 2016). These bindings would include *cues*, tasks and responses; consequently, cue repetitions are full repetition trials and cue repetition benefits are full repetition benefits.

All in all, the larger RR benefits in task repetitions when context repeated (see Fig. 1) may then be attributed to the priming of cue encoding (or to bindings that involve the cue, and not the context). Having a cue-related context thus impedes distinguishing priming of cue encoding from context-triggered retrieval of the n−1 task. Therefore, the present study aims to examine context-binding effects in the absence of possibly outcome-equivalent cue-related processes.

## The present experiment

**Fig. 2:**
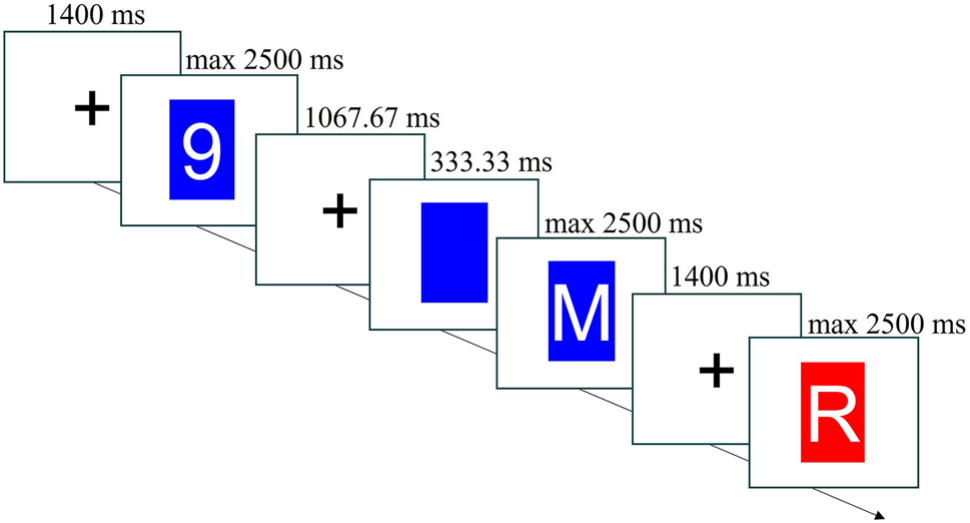
Example of the timeline of three consecutive trials. The second trial has positive SOA, and the first and the third have zero SOA. The second trial is a task switch (from the digit to the letter task) with a context repetition (blue colour patch), while the third trial is a task repetition with a context switch. The figure is not drawn to scale

In the present, preregistered experiment (http://dx.doi.org/10.23668/psycharchives.6525), we attempted to measure context-binding effects while avoiding the issue of the priming of cue encoding effects. To this aim, we removed the cues from the paradigm and used unpredictable task sequences with univalent target stimuli (e.g. Koch et al., 2023; Rogers & Monsell, 1995). Univalent targets are stimuli that only afford one of the possible tasks, thus disambiguating the relevant task in each trial. Importantly, the RR effect is typically observed also with univalent targets (e.g. Koch et al., 2023). The context was operationalized as the colour of an irrelevant coloured patch that surrounded the target. Such a patch closely resembles the context used in Experiment 1 by Benini et al. (2022a), without being a feature of the task-relevant cue (see Fig. 2).

We predicted to replicate the RR effect (preregistered hypothesis H1). Moreover, if the full repetition benefits observed in previous experiments largely depended on the priming of cue encoding, we predicted they would not emerge here. In contrast, if full repetition benefits largely depended on context binding, we predicted to replicate larger RR benefits in task repetitions when the colour of the patch repeated than when it switched (preregistered hypotheses H2a and H2b). Finding full repetition benefits without the cue would provide evidence that priming of cue encoding is not necessary for them to emerge. Thus, it would also weaken the alternative hypothesis that priming of cue encoding was responsible for the full repetition benefits of the previous studies.

The present experiment was not constructed (and it does not allow) to uncover why context transition did not usually have an effect in task switches. Nonetheless, as an exploratory question, we predicted that RR costs in task switches might be larger in context switches than in repetitions (i.e. full switch benefits, preregistered exploratory question E3). The results revealed this was not the case in the present experiment as well.

We further introduced a stimulus-onset-asynchrony (SOA) manipulation between context onset and target onset. In each trial, the colour patch could unpredictably appear before the target (positive SOA), or appear simultaneously with the target (zero SOA). We predicted positive SOA to augment the effect of context transition on the RR effect (preregistered hypothesis H3a and H3b). We derived this prediction for the following reasons (see also Benini et al., 2022a, for a similar SOA manipulation and discussion of its effects): First, it would be less likely for participants to ignore the colour patch when this is the only stimulus presented. Second, any (retrieval) process triggered by the context would not happen at the same time as any target-related process. This might give some advantage to the context-triggered retrieval to win the race against the other task-relevant processes that could otherwise mask its effects (Frings & Moeller, 2012). Third, any retrieval process triggered by the context would have more time to unfold overall.

## Materials and methods

### Participants

As per the pre-registration, we planned to recruit 96 German-speaking participants fluent in English, aged between 18 and 35 years via Prolific (https://www.prolific.co/). Our main hypothesis consisted of a three-way interaction of task transition, response transition and context transition in the RTs. We averaged the effect sizes of this three-way interaction found in the experiments of Koch et al., 2018a, b (η_*p*_^2^ = 0.25 and η_*p*_^2^ = 0.05), Kandalowski et al., 2020 (η_*p*_^2^ = 0.02 and η_*p*_^2^ = 0.04), Benini et al., 2022a (η_*p*_^2^ = 0.03 and η_*p*_^2^ = 0.12) and Benini et al., 2022b (η_*p*_^2^ = 0.03 and η_*p*_^2^ = 0.19). The resulting expected partial eta squared is a small effect: 0.09 (*d*_*z*_ = 0.31). A significant three-way interaction is equivalent to a difference of differences of differences significantly different from zero. G*Power (Faul et al., 2009) indicates that 84 participants will grant a power of 80% in a two-tailed paired sample *t* test with alpha = 0.05 and a Cohen’s *d*_*z*_ = 0.31. Therefore, 96 participants will grant power > 80%.

We collected the datasets of 97 participants and we removed one that declared having technical issues (they had to keep the alt key pressed throughout the experiment). Our sample, therefore, consisted of 96 participants (41 females, 1 “other”), who were 25.6 years old on average (*sd* = 5.12), of which nine were left-handed. Prolific calculated 21 min as the median duration of the study, for which they received £3.75. The procedure of this experiment was approved by the ethics committee of the Philosophical Faculty at RWTH Aachen University (ethics approval number: 2020_006_FB7_RWTH Aachen), and the hypotheses, the procedure and the analyses were preregistered (https://doi.org/10.23668/psycharchives.6525). The data, the analysis scripts and the experimental materials are available at: 10.23668/psycharchives.13958.

### Stimuli, tasks and responses

Instructions and stimuli were presented on a white background. The 16 targets were eight letters (A, E, O, U, K, M, T, Z) and eight digits (2, 4, 6, 8, 3, 5, 7, 9). Each target was presented in white font in the centre of the screen in Arial font size 80. Behind every target, there was the context, namely, either a red or blue rectangle (100 pixels high, 70 pixels wide), with a slightly larger height and width than the target so that it contained the target entirely (see Fig. 2), acting as the target background. The two tasks consisted of judging whether the letter was a vowel or a consonant or whether the number was odd or even. Participants responded by pressing the A or the L key on their keyboard with their left and right index fingers. The response-key mappings were counterbalanced across participants.

### Procedure

The experiment was programmed and hosted in Gorilla Experiment Builder (Anwyl-Irvine et al., 2020). Data collection took place between May and July 2022. Participants accessed the experiment via their account in Prolific exclusively using a computer or a laptop. Upon access, they were prompted to choose a quiet room and a comfortable position, and the experiment forced them to select the full-screen view. Participants read and agreed to the informed consent and data protection which they chose to read in German or English.

The instructions were presented in English and informed about the presence of the coloured rectangle, specifying that in some cases it could appear also before the target (the letter or the digit) and that the rectangle and its colour were nonetheless irrelevant to their task. Participants were also instructed to respond as fast and as accurately as possible, and that they needed to respond correctly at least 55% of the time. They were instructed to lay the fingers on the keys during the whole experiment to increase speed while they were shown a picture displaying the correct position of the fingers during the instructions and again at the beginning of each block.

Participants completed 32 training trials that included all the combinations of context (blue and red) and the 16 targets, and half of them had positive SOA. The training trials were identical to the main blocks, but they also included accuracy and speed feedback. The sentence: “Please, try to be faster!” appeared for 400 ms if neither A nor L was pressed within 2 s from the target onset. A green tick mark appeared for 400 ms if they responded correctly, and a red cross if incorrectly. In the zero-SOA condition, each trial started with a black fixation cross in the centre of the screen for 1400 ms, after which the target, either a letter or a digit, and the context appeared (see Fig. 2). In the positive SOA condition, the fixation cross stayed on the screen for 1066.67 ms, after which the context appeared alone, followed by the target after 333.33 ms. The interval between each response and the next *target* was therefore constant across the two SOA conditions (1400 ms). Context and target remained on the screen until a response was given or the deadline of 2500 ms was reached. The next-trial fixation cross appeared directly after.

After the training and after each block, participants were shown their accuracy to that point. After the training, they could re-read the introductions or start with the main experiment. Each block consisted of 64 trials where half of the trials were task repetitions and half switches. Each target was paired exactly four times with a red context, of which twice with a positive and twice with a zero SOA; the same for the blue context. The target could repeat in subsequent trials, so as not to introduce a bias in the proportion of response repetitions versus switches (Altmann, 2011). At the end of the experiment, participants responded to demographic questions and were thanked and debriefed.

### Design

The experiment had a 2 × 2 × 2 × 2 within-subjects design. The independent variables were task transition (repetition vs. switch), response transition (repetition vs. switch), context transition (repetition vs. switch), and SOA (zero, positive). The dependent variables were reaction times (RTs) and error rates (ERs). We pre-registered the main hypotheses on the RTs, and only exploratory hypotheses on the ERs since previous similar studies often failed to find any effect of context transition on ERs (Benini et al., 2022a, b).

## Results

The preregistered analyses were conducted in R Studio (R version 4.1.1, R Core Team, 2020) using the *afex* package (Ben-Shachar et al., 2020; Singmann et al., 2020) version 1.0-1 for the analyses, and the *effectsize* package (Ben-Shachar et al., 2020) version 0.8.2 to calculate *t*-tests effect sizes.

### Analyses of RTs

**Fig. 3:**
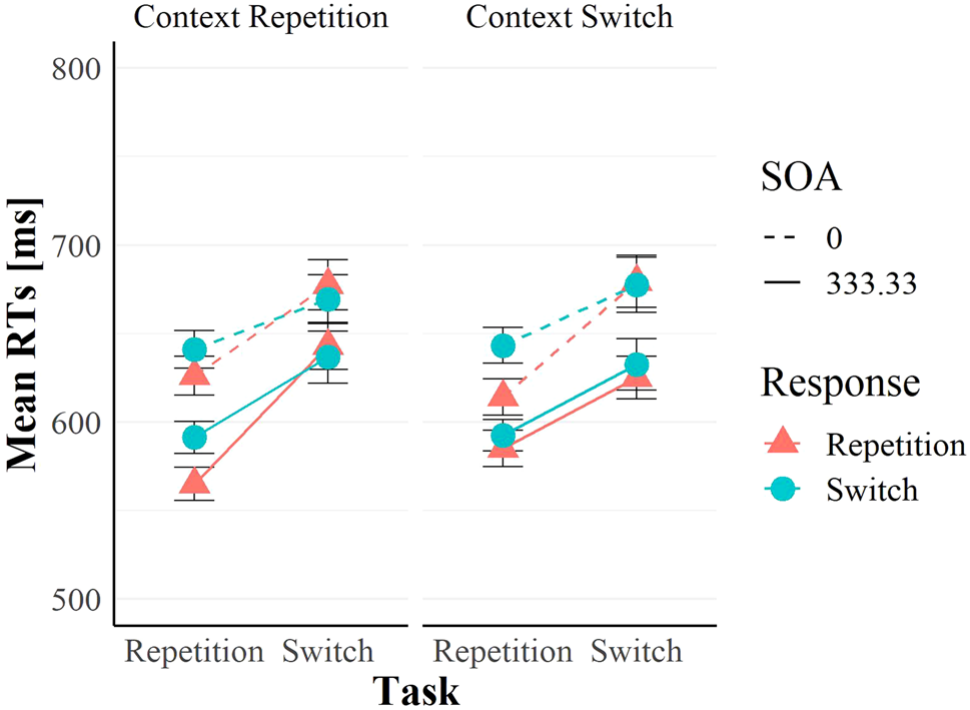
Mean RTs as a function of task transition, response transition and context transition. Vertical bars indicate the standard error of the means

**Fig. 4:**
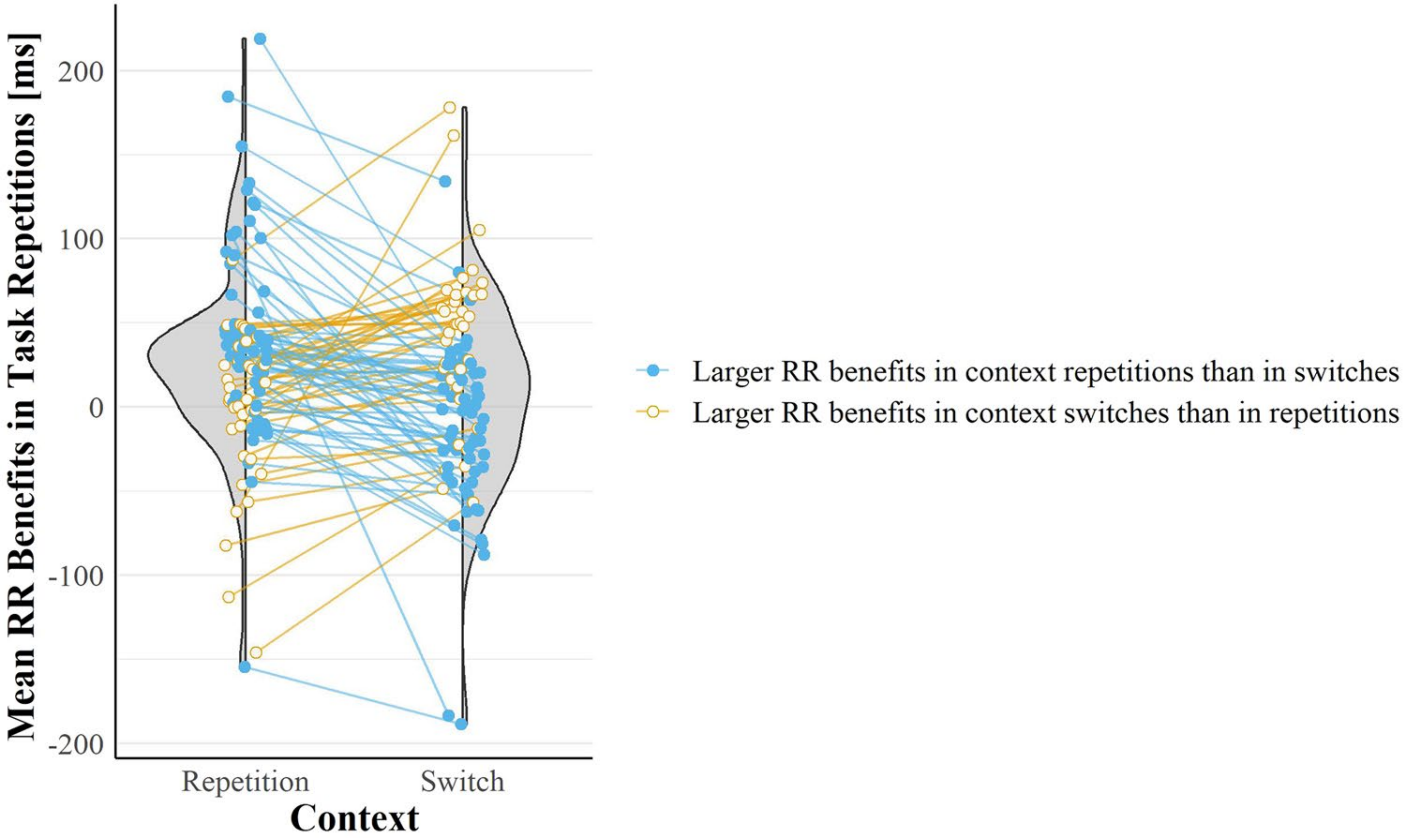
RR benefits in task repetitions in positive SOA as a function of context transition. The half violin plots show the distributions of the difference between the mean RTs in response switches minus response repetitions in task repetitions only; on the left, in context repetitions, on the right in context switches. Note that the leftward distribution has the highest density above zero, whereas the rightward around zero. Each dot in each distribution represents one participant and the two dots of each participant are linked by a line. The colour of each dot pair and the linking line reflects the slope of the line. The darkest colour [blue] and the filled dots indicate that the participant showed the same tendency as the group overall, with larger RR benefits in context repetitions than switches, the lightest colour [yellow] and the empty dots indicate the opposite. The colours and dots fillings help to highlight that most participants (62.5%) showed larger RR benefits in context repetitions than switches

For the RTs (see Figs. 3 and 4), we computed the average reaction time for each participant, and we removed those trials with RT longer than three standard deviations from the average. We further removed trials with RT shorter than 200 ms, the first trial of each block (which cannot be classified as a switch nor a repetition), and error and post-error trials. We also removed target repetition trials where the same letter or digit appeared in the subsequent trial. We, therefore, analysed 84.9% of the raw data.

The main effect of task transition was significant, *F*(1, 95) = 62.77, *p* < 0.001, η_*p*_^2^ = 0.4, η_*G*_^2^ = 0.04, indicating task repetition benefits (607 ms vs. 655 ms, in repetitions and switches, respectively). The main effect of response transition was also significant, *F*(1, 95) = 10.02, *p* = 0.002, η_*p*_^2^ = 0.1, η_*G*_^2^ < 0.01, indicating response repetition benefits (627 ms vs. 636 ms, in repetitions and switches respectively). Task transition and response transition interacted, *F*(1, 95) = 18.68, *p* < 0.001, η_*p*_^2^ = 0.16, η_*G*_^2^ < 0.01, indicating RR benefits in task repetitions (19 ms, see also Fig. 3) and small costs in task switches (− 2 ms).

The main effect of context transition was not significant, *F*(1, 95) < 0.1, *p* = 0.976, η_*p*_^2^ < 0.001, η_*G*_^2^ < 0.01, but the main effect of SOA was, *F*(1, 95) = 312.26, *p* < 0.001, η_*p*_^2^ = 0.77, η_*G*_^2^ = 0.03, indicating shorter RTs with a positive SOA (609 ms) than zero SOA (654 ms). SOA and context transition entered a significant three-way interaction with task transition, *F*(1, 95) = 19.79, *p* < 0.001, η_*p*_^2^ = 0.17, η_*G*_^2^ < 0.01. Crucially, this interaction was further qualified by a four-way interaction including response transition, *F*(1, 95) = 7.81, *p* = 0.006, η_*p*_^2^ = 0.08, η_*G*_^2^ < 0.01 (see Fig. 3). As per the preregistration, we split the dataset by SOA levels, and we found a significant three-way interaction of task transition, response transition, and context transition only with positive SOA, *F*(1, 95) = 9.30, *p* = 0.003, η_*p*_^2^ = 0.09, η_*G*_^2^ < 0.01, and not with zero SOA, *F*(1, 95) = 0.55, *p* = 0.460, η_*p*_^2^ < 0.01, η_*G*_^2^ < 0.01. The overall three-way interaction of task transition, response transition and context transition across the two levels of SOA was not significant, *F*(1, 95) = 2.48, *p* = 0.119, η_*p*_^2^ = 0.03, η_*G*_^2^ < 0.01, and a preregistered one-tailed *t*-test showed that the RR benefits in task repetitions in context repetitions (20 ms) were not significantly larger than in context switches (18 ms, *t*(95) = 0.43, *p* = 0.333, *d*_*z*_ = 0.04).

The ANOVA on zero-SOA trials revealed a main effect of task transition, *F*(1, 95) = 44.43, *p* < 0.001, η_*p*_^2^ = 0.32, η_*G*_^2^ = 0.032, and response transition, *F*(1, 95) = 6.91, *p* = 0.01, η_*p*_^2^ = 0.07, η_*G*_^2^ = 0.001, and the two variables interacted showing the usual RR effect, *F*(1, 95) = 19.81, *p* < 0.001, η_*p*_^2^ = 0.17, η_*G*_^2^ = 0.003. However, no other main effect or interaction was significant (all *F*s < 3.11, all *p*s > 0.081).

We further divided the positive-SOA trials into context repetitions and switches and we found that task transition only interacted with response transition in context repetitions, *F*(1, 95) = 11.44, *p* = 0.001, η_*p*_^2^ = 0.11, η_*G*_^2^ = 0.005, and not in switches, *F*(1, 95) < 1, *p* = 0.992, η_*p*_^2^ < 0.001, η_*G*_^2^ < 0.01 (see Fig. 3). In context repetitions, we found the usual RR benefits in task repetitions (26 ms) which became costs in task switches (− 6 ms). A non-preregistered one-tailed *t*-test on the subset of positive-SOA trials showed that RR benefits in task repetitions in context repetitions were significantly larger than in context switches (7 ms, *t*(95) = 2.3, *p* = 0.004, *d*_*z*_ = 0.28). Such RR benefits in task repetitions in positive SOA trials are depicted in Fig. 4 for each participant as a function of context transition. Figure 4 illustrates that, in positive SOA trials, 62.5% of participants indeed showed numerically larger RR benefits in task repetitions when the context repeated than when it switched. No other main effect or interaction was significant (all *F*s < 2.52, all *p*s > 0.116).

### Analyses of ERs

**Fig. 5:**
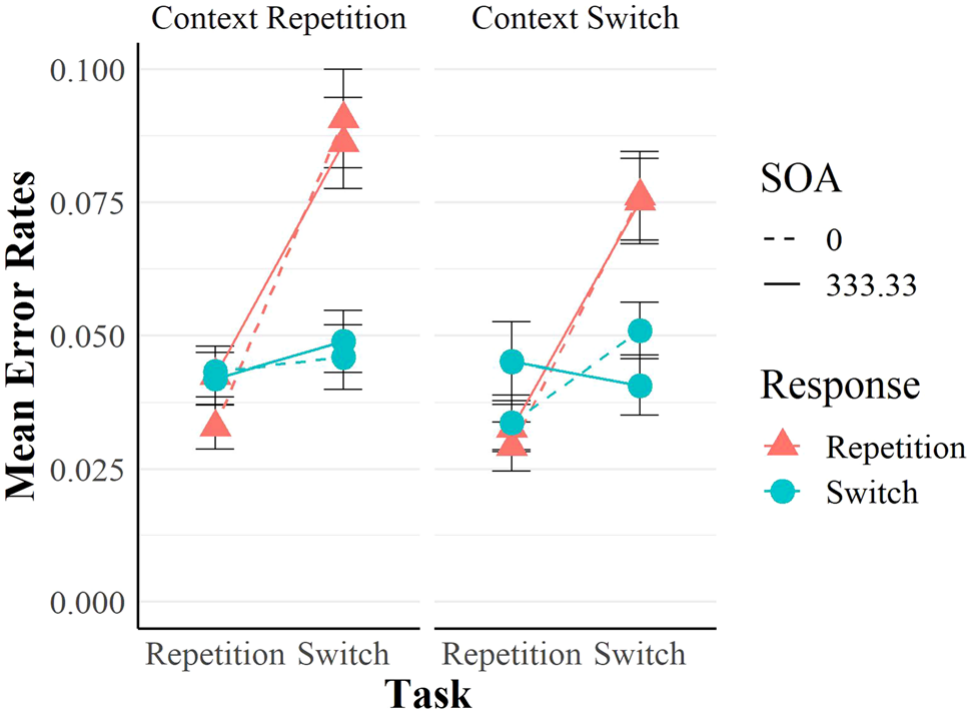
Mean ERs as a function of task transition, response transition and context transition. Vertical bars indicate the standard error of the means

For the ERs (Fig. 5), we cleaned the data as for the RTs ANOVA, but we kept error trials. Consequently, we analysed 89.5% of the raw data.

The main effect of task transition was significant, *F*(1, 95) = 42.84, *p* < 0.001, η_*p*_^2^ = 0.31, η_*G*_^2^ = 0.04, indicating task repetition benefits (0.03 and 0.06 for task repetitions and switches, respectively). The main effect of response transition was also significant, *F*(1, 95) = 18.89, *p* < 0.001, η_*p*_^2^ = 0.17, η_*G*_^2^ = 0.01, indicating general response repetition *costs* (0.06 and 0.04 in response repetition and switches, respectively). Task transition and response transition interacted, *F*(1, 95) = 48.93, *p* < 0.001, η_*p*_^2^ = 0.34, η_*G*_^2^ = 0.03, showing small RR benefits in task repetitions (0.007) and costs in task switches (− 0.03), as in the RTs.

Differently from the RTs, the main effect of context transition was significant, *F*(1, 95) = 5.69, *p* = 0.019, η_*p*_^2^ = 0.06, η_*G*_^2^ < 0.001, showing context repetition *costs* (0.05 and 0.048 in context repetitions and switches, respectively), and the main effect of SOA was not significant, 1*F*(1, 95) = 0.25, *p* = 0.618, η_*p*_^2^ < 0.001, η_*G*_^2^ < 0.001. In the ERs, the four-way interaction was not significant, *F*(1, 95) = 3.42, *p* = 0.068, η_*p*_^2^ = 0.04, η_*G*_^2^ = 0.001. No other main effect or interaction was significant (all *F*s < 2.78, all *p*s > 0.098).

## Discussion

In this experiment, we examined whether an irrelevant feature (the context) can be bound with the task and the response in task switching. We built on previous studies that examined the same question and that found encouraging results (see Fig. 1). Importantly, we intended to rule out a possible alternative explanation. Thus, we employed univalent targets so that we could remove the cue from our task-switching paradigm, to examine full repetition benefits without any confound stemming from cue-related processes.

We predicted that repeating the context, compared to switching it, would augment RR benefits in task repetitions. However, this was only the case when the context appeared already before the target presentation (i.e. positive SOA). With a positive SOA, our RTs results nicely replicate the full repetition benefits (compare Figs. 1, 3 and 4) of previous task-switching studies examining context binding (e.g. Benini et al., 2022a, b; Koch et al., 2018a, b), despite the context was not cue-related.

Such full repetition benefits observed with the positive SOA are consistent with the idea that the task, response and context become bound in each trial and that such binding can be retrieved in the following trial upon repetition of at least one feature (e.g. Altmann, 2011; Frings et al., 2020; Grange et al., 2017). Namely, when, for example, the context repeats, it retrieves the previous task and response, therefore causing full repetition benefits when both the task and the response indeed repeat, and partial repetition costs otherwise. Or, when the task repeats, it retrieves the previous context and response, therefore causing full repetition benefits when both the context (which is fully processed due to the positive SOA) and the response indeed repeat, and partial repetition costs otherwise, et cetera.

At first glance, such full repetition benefits may seem explainable by the “bypass rule” (Fletcher & Rabbitt, 1978, which is sometimes referred to as the signalling hypothesis, e.g. Koch et al., 2023; Weissman et al., 2022). Accordingly, participants learn to bypass stimulus encoding and simply repeat the response when the stimulus repeats identically and switch the response when the stimulus switches. However, evidence for the bypass rule was observed in paradigms where the task always repeated. In task switching, there are always at least two hierarchically organised levels (i.e. the task and or the cue, the stimulus category, and the stimulus) at which the current trial may switch from the previous. Thus, it is unclear whether the bypass rule would command a response switch already with a switch at only one level, or only when all levels switch. Furthermore, in its original formulation, a bypass heuristic could be ruled out given the absence of complete repetitions, since we did not analyse target repetitions. In fact, Krueger and Shapiro (1981) showed that participants adopted a bypass rule, namely tended to repeat the response to the stimulus, even when an irrelevant stimulus dimension repeated. Hence, our participants might have reacted to a context repetition with a response repetition engendering full repetition benefits. However, adopting a bypass rule also implies that response switches should be faster in context switches than in repetitions, which was not the case in our data. For example, in task repetitions, response switches were as slow in context repetitions as in context switches. Since these are both partial repetitions, binding, instead, does not predict them to be different.

To our knowledge, the literature does not provide other explanations for such a benefit for repeating an irrelevant feature in combination with relevant ones. Therefore, by weakening the hypothesis that such full repetition benefits are due to cue encoding priming (since no cue was present in this study) or by a bypass-rule strategy, we are strengthening the explanation that the context becomes bound with the task and the response, therefore impacting performance when it switches/repeats. Such short-term bindings processes may be the building blocks of longer-term learning processes (Moeller & Frings, 2017; Schmidt et al., 2020a, b) that emerge, for example, when contingencies between some features are introduced (e.g. Rubin & Koch, 2006; Waszak et al., 2003).

### The effects of an early versus late context onset

In the previous studies we cited, full repetition benefits emerged only when the context was cue-related, e.g. cue colour, and not when it was target-related, e.g. target colour (Benini et al., 2022a). Such observation suggested that cue-encoding priming could have contributed to such a pattern. In the present study, only when SOA was positive, that is, when the context appeared already before the target, we observed full repetition benefits, and not when context and target appeared together. We indeed predicted a positive SOA to augment the full repetition benefits. In the introduction (see also the discussion of Benini et al., 2022a, p. 14), we had already proposed three explanations for why an early context onset may yield full repetition benefits. Namely, it guarantees more time for the context to be encoded and processed, it increases the difficulty of ignoring the context since it is presented in isolation, and it prevents target-triggered processes from masking context-triggered retrieval.

However, we did not expect positive SOA to be a *necessary* condition for such full repetition benefits to emerge. Yet, combining the results from the previous studies and the present study we derive that the necessary condition for full repetition benefits to emerge seems not for the context to be cue-related, but for the context to appear early enough in the trial timeline.

Furthermore, a positive SOA implied that the context was the first stimulus of that trial, immediately establishing a relative continuity (when the context repeated) versus discontinuity (when the context switched) with the previous trial. In other words, when context is the first stimulus it might set the “event boundaries” (Zacks & Swallow, 2007) between the previous and the current trial. Namely, if the context repeats, participants may maintain active the last task set and response, hence showing task and response repetition benefits. On the contrary, if the context switches, participants may segment the previous trial from the current trial, diminishing the probability that the previous trial is retrieved, hence showing smaller task and response repetition benefits. Interestingly, the same argument holds for cue repetition benefits (see The Priming of Cue Encoding section in the introduction). Namely, the same task set might be more easily maintained active upon a cue repetition than a switch, hence causing larger task repetition benefits in cue repetitions than in cue switches.

A similar explanation was suggested in the context of negative priming paradigms by Qiu et al. (2023). They found that repeating a context from a pre-prime to a prime trial reduced the probability for the prime to be retrieved in the probe, as if such a common context blurred the borders between the pre-prime and the prime in working memory. Relatedly, Wang and Egner (2022) provided evidence that repeating a *task* across several trials may act as an event-integration factor. Participants performed a task-switching experiment whose mini-runs were interleaved with questions about the presented stimuli. After each mini-run of 30 trials, they asked participants to recall how many trials apart two stimuli were presented, and in which order. They found that switching the task between two trials, compared to repeating it, increased the perceived distance between the two events and increased the confusion between their order.

## Conclusion

The present study showed that priming of cue-encoding cannot be the critical factor responsible for full repetition benefits to emerge in a task-switching paradigm with an irrelevant context. These data, therefore, reinforce the hypothesis that such full repetition benefits stem from binding and retrieval processes that take place on a trial-by-trial basis and involve both task-relevant (the task and the response) and task-irrelevant features (the context).

## Data Availability

The raw and preprocessed data, the code used to run the analyses and some study materials are made available online at 10.23668/psycharchives.13958. It is possible to get access to the full experiment workflow, which was built in Gorilla Experiment Builder, upon request to the correspondence author.

## Footnotes

1 Thus, a positive SOA speeded responses but did not improve accuracy. Arguably, it might have acted as an alerting signal that increased participants’ readiness and thus RTs, independently from whether context repeated or switched.
